# Fourth-Of-July Tetanus

**Published:** 1901-06-29

**Authors:** 


### FOURTH-OF-JULY TETANUS.

Once a year the inhabitants of the United States are wont to celebrate the anniversary of the Declaration of Independence by the firing off of many guns. Incidentally the event is marked by the blowing off of many fingers, and it is noted as a curious fact that a very considerable number of these injuries are followed by tetanus. Fourth-of-July tetanus has come to be a phrase. Dr. Gideon Wells,[1] writing on the subject, says that from June 25th to July 14th, 1900, there were reported to the coroner of Cook County 29 deaths due to tetanus, all but two of which were to be attributed to wounds from explosives used in demonstrating Independence Day, for the Chicago youth distributes his abundant patriotism over several weeks. The 27 victims were all boys, and with one exception had received their injuries from the discharge of blank cartridges, the exception being a wound from a toy cannon. The injuries were not due to bursting of the weapons, but to inexperience in their use. The question arises, why should tetanus be such a frequent result of Fourth-of-July wounds? After a careful examination of the various ways in which infection might have come about, Dr. Wells concludes that the cause is the dirt on the hands by which the wound becomes infected, it being often carried deeply into the wound by the wad and there retained from the wound not being thoroughly laid open for drainage. When the average patriot goes out to celebrate the Fourth of July he usually betakes himself, says Dr. Wells, to some vacant lot or to the streets of his native town, and after a few hours of energetic celebrating his hands are covered with several layers of dirt; and as street dirt and stable manure are the special habitat of the tetanus bacillus, the wound is at once inoculated. The same thing has again and again been noticed in regard to injuries on the football field, which are followed by tetanus in a far larger proportion than is the case with wounds arising under other circumstances. Dr. Wells urges that in Fourth-of-July injuries the greatest pains should be taken to open up the recesses of the wounds and extract the bits of wadding, &c.; but he also insists on the desirability of injecting tetanus antitoxin as a prophylactic measure, in view of the known risk of the supervention of tetanus in such cases.

[1] New York Med. News.

